# Supplementary material for: Physiological responses and adaptations to exercise training in people with or without chronic obstructive pulmonary disease: protocol for a systematic review and meta-analysis
Source: BMJ Open. 2022 Sep 19;12(9):e065832. doi: 10.1136/bmjopen-2022-065832 (PMC9486278; doi:10.1136/bmjopen-2022-065832)
Supplement: Supplementary data [file bmjopen-2022-065832supp002.pdf]

Database:

Ovid MEDLINE(R) and Epub Ahead of Print, In-Process, In-Data-Review & Other Non-Indexed Citations, Daily and Versions(R) <1946 to February 15, 2022>

Search as per 2022-02-15:

1. exp Pulmonary Disease, Chronic Obstructive/ (62214)
2. COPD.mp. (53231)
3. Chronic Obstructive Pulmonary Disease.mp. (55582)
4. Chronic Obstructive Airway Disease.mp. (309)
5. Chronic Obstructive Lung Disease.mp. (4419)
6. Airflow Obstruction$, Chronic.mp. (11)
7. Airflow limitation.mp. (3405)
8. exp Exercise/ (226020)
9. exp Exercise Therapy/ (58389)
10. Exercise Program.mp. (9485)
11. High-Intensity Interval.mp. (3443)
12. exp High-Intensity Interval Training/ (1691)
13. Interval Exercise.mp. (862)
14. Intermittent Exercise.mp. (1174)
15. Intermittent Training.mp. (191)
16. Continuous Exercise.mp. (788)
17. Continuous Training.mp. (1403)
18. Moderate-Intensity Continuous Exercise.mp. (173)
19. Sprint interval training.mp. (384)
20. Physical Exercise.mp. (18246)
21. Pulmonary Rehab*.mp. (4422)
22. Aerobic Exercise.mp. (11132)
23. Anaerobic exercise.mp. (622)
24. Exercise Training.mp. (19075)
25. exp Endurance Training/ (464)
26. Strength Training.mp. (5996)
27. exp Resistance Training/ (10819)
28. NMES.mp. (1389)
29. Neuromuscular Stimulation.mp. (525)
30. Inspiratory Muscle Training.mp. (831)
31. Cycling.mp. (70584)
32. Tai Chi.mp. (2083)
33. exp Tai Ji/ (1305)
34. exp Walking/ (62002)
35. 1 or 2 or 3 or 4 or 5 or 6 or 7 (97573)
36. 8 or 9 or 10 or 11 or 12 or 13 or 14 or 15 or 16 or 17 or 18 or 19 or 20 or 21 or 22 or 23 or 24 or 25 or 26 or 27 or 28 or 29 or 30 or 31 or 32 or 33 or 34 (374060)
37. 35 and 36 (6309)
38. exp animal/ not humans.sh. (4959724)
39. 37 not 38 (6264)
