# Supplementary material for: Physiological responses and adaptations to exercise training in people with or without chronic obstructive pulmonary disease: protocol for a systematic review and meta-analysis
Source: BMJ Open. 2022 Sep 19;12(9):e065832. doi: 10.1136/bmjopen-2022-065832 (PMC9486278; doi:10.1136/bmjopen-2022-065832)
Supplement: Supplementary data [file bmjopen-2022-065832supp003.pdf]

Modified 14-item Black and Downs checklist

1. Is the hypothesis/aim/objective of the study clearly described?
2. Are the main outcomes to be measured clearly described in the introduction or methods section?
3. Are the characteristics of the patients included in the study clearly described?
4. Are the distributions of principal confounders in each group of subjects to be compared clearly described?
5. Are the main findings of the study clearly described?
6. Does the study provide estimates of the random variability in the data for the main outcomes?
7. Have the characteristics of patients lost to follow-up been described?
8. Have actual probability values been reported for the main outcomes? (except where the probability value is <0.001)
9. Were the subjects asked to participate in the study representative of the entire population from which they were recruited?
10. Were those subjects who were prepared to participate representative of the entire population from which they were recruited?
11. Were the statistical tests used to assess the main outcomes appropriate?
12. Were the main outcome measures used accurate (valid and reliable)?
13. Were losses of patients to follow-up taken into account?
14. Did the study have sufficient power to detect a clinically important effect/difference where the probability value for a difference being due to chance is less than 5%?
