# Supplementary material for: Physiological responses and adaptations to exercise training in people with or without chronic obstructive pulmonary disease: protocol for a systematic review and meta-analysis
Source: BMJ Open. 2022 Sep 19;12(9):e065832. doi: 10.1136/bmjopen-2022-065832 (PMC9486278; doi:10.1136/bmjopen-2022-065832)
Supplement: Author's manuscript [file bmjopen-2022-065832.draft_revisions.pdf]

BMJ Open is committed to open peer review. As part of this commitment we make the peer review history of every article we publish publicly available.

When an article is published we post the peer reviewers' comments and the authors' responses online. We also post the versions of the paper that were used during peer review. These are the versions that the peer review comments apply to.

The versions of the paper that follow are the versions that were submitted during the peer review process. They are not the versions of record or the final published versions. They should not be cited or distributed as the published version of this manuscript.

BMJ Open is an open access journal and the full, final, typeset and author-corrected version of record of the manuscript is available on our site with no access controls, subscription charges or pay-per-view fees (http://bmjopen.bmj.com).

If you have any questions on BMJ Open's open peer review process please email info.bmjopen@bmj.com

BMJ Open

Physiological Responses and Adaptations to Exercise Training in People with or without Chronic Obstructive Pulmonary Disease: Protocol for a Systematic Review and Meta-Analysis

Journal:	*BMJ Open*
Manuscript ID	bmjopen-2022-065832
Article Type:	Protocol
Date Submitted by the Author:	20-Jun-2022

Complete List of Authors:	Jakobsson, Johan; Umea University, Community Medicine and Rehabilitation, Physiotherapy<br>De Brandt, Jana; Umea University, Community Medicine and Rehabilitation, Physiotherapy<br>Nyberg, André; Umea University, Department of Community Medicine and Rehabilitation, Physiotherapy

Keywords:	Chronic airways disease < THORACIC MEDICINE, Respiratory physiology < THORACIC MEDICINE, Physiology < NATURAL SCIENCE DISCIPLINES, SPORTS MEDICINE

SCHOLARONE™
Manuscripts

Physiological Responses and Adaptations to Exercise Training in People with or without Chronic Obstructive Pulmonary Disease: Protocol for a Systematic Review and Meta-Analysis

Jakobsson, J*[1]., De Brandt, J[1]., Nyberg, A[1].

***Corresponding author:** Johan Jakobsson, johan.jakobsson@umu.se, +46 70 271 46 01

Author affiliations

1 Section of Physiotherapy, Department of Community Medicine and Rehabilitation, Faculty of Medicine, Umeå University, Linneaus Väg 8, 907 36 Umeå

Johan Jakobsson: johan.jakobsson@umu.se

Jana De Brandt: jana.de.brandt@umu.se

Andre Nyberg: andre.nyberg@umu.se

Word count: 4000 (Background to Amendments)

Key words: COPD; exercise training; pulmonary rehabilitation; acute exercise

For peer review only

ABSTRACT

Introduction

Exercise training is a cornerstone in managing chronic obstructive pulmonary disease (COPD), leading to several physiological adaptations including, but not limited to, structural and muscular alterations, increased exercise capacity, and improved cognitive function. Still, it is not uncommon that the physiological acute response to an exercise session and extent of chronic adaptations to exercise training are altered compared to people without COPD. To date, potential differences in physiological acute responses and chronic adaptations in people with or without COPD are not fully understood, and results from individual studies are contradictory. Therefore, the aim of the current study is to synthesize and compare the physiological acute responses and chronic adaptations to exercise training in people with COPD compared to people without COPD.

Methods and analyses

A systematic review of randomised controlled trials (RCT), non-randomised studies of interventions (NRSI), and cross-sectional studies (CSS) will be conducted. A comprehensive search strategy will identify relevant studies from MEDLINE, Scopus, CINAHL, SPORTDiscus, CENTRAL and Cochrane Airways Trials Register databases. Studies including adults with and without COPD will be considered. Outcomes will include cardiorespiratory, muscular, and cognitive function, intramuscular adaptations, lung volumes, and cardiometabolic responses. The protocol is reported according to the Preferred Reporting Items for Systematic Reviews and Meta-Analyses Protocols. Further guidance from Cochrane Methodological Expectations of Cochrane Intervention Reviews has been obtained. Risk of bias assessment will be conducted using Cochranes Risk-of-Bias 2 tool (RCTs), Risk-of-Bias in Non-Randomized Studies Tool (NRSIs), and Downs and Black checklist (CSSs). Meta-analyses will be conducted when appropriate, supplemented with a systematic synthesis without meta-analysis.

Ethics and dissemination

As this study is a systematic review, ethical approval is not required. The final review results will be submitted for publication in a peer-reviewed journal and presented at international conferences.

PROSPERO registration number: CRD42022307577

For peer review only

Article Summary

Strengths and limitations of this study

- This protocol is reported according to the Preferred Reporting Items for Systematic Reviews and Meta-Analyses Protocols.
- The protocol includes a comprehensive and peer-reviewed search strategy and broad inclusion criteria to allow for a comprehensive synthesis of available evidence.
- This systematic review will be the first to explore the differences in physiological acute responses and chronic adaptations to exercise training in people with COPD compared to people without COPD.
- Exclusion of literature written in languages not known by the research group might leave relevant literature out of the systematic review.
- The systematic review will be reported in line with the Preferred Reporting Items for Systematic Reviews and Meta-Analyses and systematic synthesis without meta-analysis guidelines.

For peer review only

For peer review only - http://bmjopen.bmj.com/site/about/guidelines.xhtml

BACKGROUND

Chronic obstructive pulmonary disease (COPD) is a chronic respiratory disease characterized by a persistent and progressive airflow limitation, causing breathlessness, productive coughing, fatigue, and recurrent chest infection.[1] COPD is highly prevalent, with the global prevalence among individuals aged ≥40 years being 11.7%.[2] The World Health Organization estimated that COPD is the third leading cause of death with 3.23 million deaths in 2019,[3] which is expected to increase over the next 40 years.[4 5]

Although COPD is primarily a respiratory disease, it is best understood as a systemic disease with several extrapulmonary manifestations.[6] Most people (40-98%[7-9]) with COPD have comorbidities such as cardiovascular disease, diabetes or metabolic syndrome, muscle atrophy, cognitive dysfunction, or muscle dysfunction[6 10 11] that directly and substantially impact the disease. On average, a person with COPD suffers from four extrapulmonary manifestations[12] and they are observed across the entire spectrum of airflow limitation severity.[13] Having one or more comorbidities is associated with more hospitalizations and increased mortality.[14] Still, extrapulmonary manifestations are an overlooked aspect of COPD that is not dealt with optimally even though they negatively impact important clinical outcomes independent of the degree of lung impairment.[10 15] For instance, reductions in limb muscle strength and endurance capacity are intimately linked with a reduced quality of life,[16] exercise intolerance,[17-19] greater healthcare utilization,[20] decreased ability to perform daily activities,[21] and increased mortality.[22 23] For example, quadriceps muscle atrophy is associated with a 4-fold increase in mortality even after adjusting for age, sex, and lung function.[10] At the same time, people with coexisting COPD and cognitive dysfunction have a mortality rate nearly 3-times as high compared to those having each condition alone.[15]

Importantly, and similarly, as for the healthy population and most chronic diseases,[24] exercise training is one of the cornerstones in treating extrapulmonary manifestations such as decreased muscle, cardiovascular or cognitive function in COPD according to international guidelines.[1 14 25 26] Regular exercise training in people with COPD can increase cardiorespiratory fitness, partly due to increased mitochondrial density and oxidative enzyme activity.[27] Importantly, exercise training has also been shown to reduce dyspnea and fatigue during daily life activities, decrease anxiety and depression, and improve health-related quality of life.[28] Even though it is evident that exercise training improves multiple extrapulmonary manifestations in people with COPD, the physiological acute response and extent of chronic adaptations, however, are repeatedly reported to be altered compared to healthy individuals without COPD and vary amongst people with COPD[29]. Recently, it was showed that the response to aerobic training concerning mitochondrial function is blunted in COPD compared to matched individuals without COPD.[30] However, a blunted response to exercise training is not a universal finding. For instance, Rabinovich et al. [31] reported a difference in oxidative stress between people with COPD and those without COPD following high-intensity training, while Puente-Maestu[32] did not. Costes et al.,[33] found no change in capillary-to-fiber ratio and mean fiber size in people with COPD after multidisciplinary exercise training, while Gouzi et al.[34] showed similar increases in those with and without COPD following endurance training. It has been reported that the mechanical efficiency and exercise capacity in the upper limbs is reduced in people

For peer review only - http://bmjopen.bmj.com/site/about/guidelines.xhtml

with COPD compared to people without COPD,[35] while others reported similar levels of the outcomes between people with and without COPD.[36] Thus, although multiple studies have been conducted, these seemingly contradictory findings highlight the need for a systematic comparison of studies that have evaluated acute responses and chronic adaptations to exercise training in people with COPD compared to individuals without COPD. Increased knowledge about altered and even blunted acute and chronic responses to exercise training in COPD is needed to better tailor exercise training in people with COPD.

Aim and objectives

This systematic review and meta-analysis aim to synthesize the acute physiological responses and chronic adaptations to exercise training in people with COPD compared to healthy controls. Specifically, the review will address the question: Are there differences in acute physiological responses and chronic adaptations to exercise training in people with or without COPD?

The primary objective is to compare the chronic physiological adaptations to exercise training in people with or without COPD performing the same exercise intervention. The secondary objective is to compare the acute physiological response to exercise training in people with or without COPD performing the same exercise session.

METHODS

The study protocol of this systematic review is reported in line with the Preferred Reporting Items for Systematic Reviews and Meta-Analyses Protocols (PRISMA-P) guideline (see supplemental appendix 1).[37] The systematic review will be reported according with PRISMA 2020 guidelines.[38] The systematic review protocol is registered in the International Prospective Register of Systematic Reviews (PROSPERO) on March 27, 2022 (CRD42022307577). Methodological guidance for this systematic review protocol has been obtained from Cochrane Airways, based on Cochrane Methodological Expectations of Cochrane Intervention Reviews (MECIR).

Eligibility criteria

Studies will be selected according to the criteria outlined below.

Types of study designs

Randomized controlled trials (RCTs), controlled non-randomized studies of interventions (NRSI), cluster trials and prospective comparative cohort studies will be included to synthesize chronic physiological adaptations to exercise training in people with COPD in comparison to people without COPD. Studies presenting a cross-sectional comparison of acute physiological responses to an exercise training session will be included to compare acute physiological responses between people with and without COPD. Retrospective studies, case reports, case series and opinion pieces will be excluded.

Types of participants

Any study including adults ≥18 years of age with a diagnosis of COPD and a group of individuals without COPD will be considered. The COPD diagnosis should be confirmed by spirometry following international guidelines.[1] There will be no eligibility criteria for sex or

For peer review only - http://bmjopen.bmj.com/site/about/guidelines.xhtml

ethnicity. Studies including participants with COPD and comorbidities will not be excluded, but COPD should be the primary disease.

Types of intervention

Any type of acute exercise training session and exercise training interventions will be considered as long as data can be separated for and compared between people with COPD and those without COPD. For example, but not limited to endurance training, interval training, resistance/strength training, neuromuscular electrical stimulation (NMES), or inspiratory muscle training (IMT). For chronic adaptations to exercise training, interventions with a duration of fewer than three weeks or 12 sessions will be excluded.

Types of outcome measures

Studies will not be excluded on the basis of outcomes. The following outcomes are of interest, including but not limited to:

- Cardiorespiratory function
  - Maximal exercise performance: peak oxygen consumption (VO_2peak) and max aerobic power during incremental exercise test.
  - Exercise capacity: e.g. 6-minute-walk-test, endurance shuttle walk test, constant-work rate cycle test, step-test.
- Cardiorespiratory and metabolic responses during exercise: e.g. heart rate, peripheral capillary oxygen saturation, oxygen consumption, carbon dioxide production, concentration of blood lactate, markers of mitochondrial biogenesis, myogenesis, neuroprotection, inflammation and oxidative stress.
- Muscle function:
  - Muscle strength: Maximal muscle force (e.g., maximal voluntary contraction), isokinetic or isometric (peak torque), and isotonic strength (1-repetition maximum).
  - Muscle power: Repeated maximal contractions at a submaximal load (i.e., to generate an increase in contractile force over a certain unit of time).
  - Muscle endurance: Change in muscle force over time (e.g., time to failure when maximal or submaximal static or dynamic contractions are performed).
  - Functional performance: e.g. 5-time sit-to-stand test, 30-sec sit-to-stand and 1-minute sit-to-stand test.
- Intramuscular adaptations: e.g. Mitochondrial function and markers, markers of oxidative stress and inflammation, enzyme activity, fibre type proportion, fibre size, and capillarization.
- Lung volumes: e.g. Tidal volume, minute ventilation, breathing frequency, ventilation to maximum voluntary ventilation ratio, dynamic hyperinflation, end-expiratory lung volume, end-inspiratory lung volume, and inspiratory capacity.
- Cognitive function: Global cognitive tests (e.g. Montreal Cognitive Assessment, Mini-Mental State Examination, General Practitioner Assessment of Cognition, Mini Cog Test).

Timing

Only before and after intervention data will be included for intervention studies with more than two time points of assessments.

For peer review only - http://bmjopen.bmj.com/site/about/guidelines.xhtml

For peer review only

Setting
There will be no exclusion by type of setting.

Timespan
All articles from inception to the start of the literature search will be considered. Any restriction of time period is not deemed relevant.

Language
English, Swedish, Norwegian, Danish, French and Dutch literature will be considered.

SEARCH METHODS FOR IDENTIFICATION OF STUDIES

Electronic searches

Relevant studies will be identified from (database inception to date):

- MEDLINE (Ovid SP interface)
- Scopus
- CINAHL and SPORTDiscus (EBSCO interface)
- Cochrane Central Register of Controlled Trials (CENTRAL)
- Cochrane Airways Trials Register.

Search for grey literature, registered trials and ongoing studies will be performed, from inception of data base to date, through but not limited to:

- World Health Organization International Clinical Trials Registry Platform (ICTRP)
- International Standard Randomised Controlled Trial Number (ISRCTN)
- National Institutes of Health Clinical Trials Database (ClinicalTrials.gov)

Corresponding authors or principal investigators of identified unpublished and studies in progress will be contacted to establish whether published literature was missed.

Other resources

Relevant conference proceedings will be searched, and if required, we will contact authors of identified trials for unpublished studies or missing information. If sufficient data is provided, conference abstracts will be included.

The reference list of included studies identified through the search will be hand-searched for additional studies. Other systematic reviews on the topic will also be checked for further studies. The 'related articles' function in PubMed will be used on included studies or relevant reviews.

The Cochrane Database of Systematic Reviews and PROSPERO have also been searched for existing or ongoing reviews on the topic. No previous or ongoing systematic review was identified. We will search PubMed for errata or retractions from included studies published in full text and report which date this was done within the review.

Search strategy

The search strategy is based on the Cochrane Airways Group Register of Trials guidelines. It has been developed with the assistance of a health science librarian and peer-reviewed by experts in exercise training, pulmonary rehabilitation, and lung diseases. To ensure literature saturation, comprehensive searches will be constructed of both index terming (MeSH terms),

For peer review only - http://bmjopen.bmj.com/site/about/guidelines.xhtml

free text terms, and synonyms. No language restriction will be imposed on the search. A draft of the search strategy for MEDLINE (OVID interface) is presented in online supplemental appendix 2. The final MEDLINE search strategy will be adapted to other databases' and trial registries syntax and subject headings.

Data collection and analysis

Study records

The results from the literature searches will be uploaded to Covidence (Covidence, Melbourne, Australia). This internet-based software program facilitates the management of studies, including removing duplicates and collaboration among reviewers during the study-selection process. The research team will develop, test, and refine screening questions and forms for assessments based on the eligibility criteria. Members of the review team not familiar with Covidence will be trained in the software prior to the start of the review. The search process will be documented, including:

- the name of the database searched
- the name of the database provider/system used
- the date when the search was run
- the years covered by the search
- the search terms used, hits per search term, and number of articles retrieved

Selection process

Two review authors (JJ and JDB) will screen the titles and abstracts of the search results independently and code them as 'retrieve' (eligible or potentially eligible/unclear) or 'do not retrieve'. Then, we will retrieve the full-text study reports of all potentially eligible studies. Two review authors (JJ and JDB) will independently screen them for inclusion, recording the reasons for exclusion of ineligible studies. If needed, additional information from study authors will be sought using e-mail to resolve questions about eligibility. We will resolve any disagreement through discussion or, if required, we will consult a third review author (AN), and a majority rule will be used. Neither of the review authors will be blind to the journal titles, the study authors, or institutions. Special attention will be given to identifying and excluding duplicates and collating multiple reports of the same study. Rather than each report, each study is the unit of interest in the review. We will record the selection process in sufficient detail to complete a PRISMA flowchart diagram. The inter-assessor agreement will be assessed using Kappa statistics.

Data collection process

Two reviewers (JJ and JDB) will use a standardized data extraction form to extract data independently from full texts of all included studies. The form will be pilot-tested on two to three potentially eligible articles and reviewers trained in using the form. Disagreement will be solved by consensus. When disagreement cannot be resolved by consensus, a third reviewer (AN) will be consulted, and a majority rule will be used. In the absence of complete descriptions of outcomes and effect estimates, authors will be asked for additional information or missing

For peer review only - http://bmjopen.bmj.com/site/about/guidelines.xhtml

data using e-mail. All data will be double-checked with the included studies by a third reviewer (AN). The following study characteristics will be extracted:

- Study: author(s) name, title, publication year
- Methods: study design, total duration of study, number of study centers (if multicenter), study setting
- Participants: sample size, mean age, age range, sex, inclusion and exclusion criteria, baseline lung function. For COPD group: severity of condition.
- Intervention: type of exercise training, intensity, duration, frequency and progression principles
- Outcomes: Data will be sought on the following outcomes: Maximal exercise performance, exercise capacity, cardiorespiratory and metabolic responses during exercise, muscle strength, muscle power, muscle endurance, functional performance, intramuscular adaptations, and lung volumes. Chronic adaptations following an exercise intervention period in the abovementioned outcomes are primary outcomes since effects on these outcomes are vital objectives in international COPD treatment guidelines [6 39 40]. In addition, acute responses from a single exercise session in the abovementioned outcomes are secondary outcomes. The "Type of outcome measures" section includes detailed descriptions of selected outcomes. Change from baseline will be the preferred metric for outcomes.

One review author (JJ) will transfer data into a Review Manager software (Revman, Cochrane Collaboration, Oxford, UK). We will double-check that data are entered correctly by comparing the data presented in the systematic review with the study reports. A second review author (JDB) will spot-check study characteristics for accuracy against the study report.

Risk of bias assessment

The risk of bias of eligible studies will be assessed independently by two authors (JJ and JDB) using appropriate tools. We will resolve any disagreements by discussing or involving another author (AN). No study will be excluded due to poor quality, but sensitivity analyses excluding poor-quality studies will be performed.

RCTs will be assessed using the criteria outlined in *Cochrane Handbook for Systematic Reviews of Interventions* Version 6.2, using the Risk of Bias Tool 2 (RoB 2)[41 42]. We will assess the risk of bias of a trial according to the following domains:

1. bias arising from the randomisation process;
2. bias due to deviations from intended interventions;
3. bias due to missing outcome data;
4. bias in measurement of the outcome; and
5. bias in selection of the reported result.

Signalling questions in the RoB 2 tool will provide the basis for the tool's domain-level judgements about the risk of bias. To manage the assessment of bias of included studies we will

For peer review only - http://bmjopen.bmj.com/site/about/guidelines.xhtml

For peer review only

use templates and spreadsheets of the RoB 2 tool.[42] We will reach a risk of bias judgement and assign one of three levels to each domain and an overall judgement per study:

- low risk of bias;
- some concerns; or
- high risk of bias.

The Risk of Bias in Non-randomized Studies of Interventions (ROBINS-I)[43] will be used for assessing the quality of NRSIs. This is the recommended tool by Cochrane Scientific Committee to assess the RoB in NRSIs. The ROBINS-I tool is similar to the RoB 2 tool and comprises a series of signalling questions assessing the bias in seven domains:

1. bias due to confounding;
2. bias in selection of participants into the study;
3. bias in classification of interventions;
4. bias due to deviations from intended interventions;
5. bias due to missing data;
6. bias in measurement of outcomes; and
7. bias in selection of the reported result

Important confounding domains that are prognostic factors for the outcomes of interest are: extent of matching between the groups with and without COPD, especially regarding daily physical activity (PA) and any differences in physical capacity.

We will reach a risk of bias judgement and assign one of the five levels to each domain and an overall judgement per study:

- low risk of bias;
- moderate risk of bias;
- serious risk of bias;
- critical risk of bias; or
- no information (on which to base a judgement about risk of bias)

For cross-sectional studies, the risk of bias will be assessed using a modified version of the checklist by Downs and Black[44]. This checklist has been recommended for assessing the quality for RCT and NRSI, as well as cross-sectional studies.[45] The 14 most relevant items will be used. The 14 items are appropriate for NRSI and have previously been used in studies within the field.[46] The psychometric properties of the checklist have been validated and reported elsewere[44]. The 14 items are yes (1 point) or no (0 point) questions resulting in a score between 0 and 14 where higher scores indicate higher methodological quality. The following cut-points will be used to categorize studies by quality: "Excellent" (13-14 points), "good" (10-12 points), "fair" (8-9 points) or "poor" ≤7 points). The 14 items are:

1. Is the hypothesis/aim/objective of the study clearly described?

For peer review only - http://bmjopen.bmj.com/site/about/guidelines.xhtml

2. Are the main outcomes to be measured clearly described in the introduction or methods section?
3. Are the characteristics of the patients included in the study clearly described?
4. Are the distributions of principal confounders in each group of subjects to be compared clearly described?
5. Are the main findings of the study clearly described?
6. Does the study provide estimates of the random variability in the data for the main outcomes?
7. Have the characteristics of patients lost to follow-up been described?
8. Have actual probability values been reported for the main outcomes? (except where the probability value is <0.001)
9. Were the subjects asked to participate in the study representative of the entire population from which they were recruited?
10. Were those subjects who were prepared to participate representative of the entire population from which they were recruited?
11. Were the statistical tests used to assess the main outcomes appropriate?
12. Were the main outcome measures used accurate (valid and reliable)?
13. Were losses of patients to follow-up taken into account?
14. Did the study have sufficient power to detect a clinically important effect/difference where the probability value for a difference being due to chance is less than 5%?

Synthesis

Data will be meta-analysed in RevMan, and will be presented in forests plots and a table providing a summary of study characteristics and the findings. Other computer software, such as Jamovi might also be used for supplemental analyses. For data that cannot be meta-analysed, i.e., due to a lack of data to calculate effect sizes or too diverse study characteristics, a systematic synthesis without meta-analysis (SWiM)[47] will be presented in the text. The SWiM will be conducted according to the PRISMA and SWiM[47] reporting guidelines. The SWiM guideline is a nine-item checklist to promote transparent reporting of systematic reviews.[47]

Meta-analyses will be undertaken where meaningful; if the participants, intervention, and study design are similar enough for pooling to make sense. We anticipate only continuous data to be reported for the outcomes of interest. Continuous data will be analysed as the mean difference (MD) where outcome data are reported on a uniform scale or standardized mean difference (SMD), when different metrics are used but deemed clinically homogenous.

A random-effects model will be used to allow for random error and inter-study variability. Meta-analyses will be based on change from baseline rather than post-intervention values when available. This will be done according to recommendations in *Cochrane Handbook for Systematic Reviews of Interventions* Version 6.2, section 6.5.2.[41] If needed, additional information from study authors will be sought using e-mail.

Instead of completer or per-protocol analyses, we will use intention-to-treat where they are reported (i.e. those where data have been imputed for randomly assigned participants but did

For peer review only

not complete the study). In addition, where multiple trial arms are reported in a single study, we will include only relevant ones.

Assessment of heterogeneity

We will use the I^2 and τ^2 statistics to measure heterogeneity between the studies in each analysis. If we identify substantial heterogeneity we will report it and explore the possible causes by prespecified subgroup analysis.

Assessment of reporting biases

If we are able to pool more than ten studies we will create and examine the asymmetry of a funnel plot and perform Egger's test to explore possible small study and publication biases. The possible influence of small-study/publication biases on review findings will be a part of our 'Risk of bias' assessment and GRADE assessments of the quality of the evidence.

Subgroup analysis and investigation of heterogeneity

We plan to carry out the following subgroup analyses if adequate data can be obtained:

1. extent of matching on PA (matched with any type of method compared to not matched);
2. duration of intervention ≥ 20 sessions compared to < 20 sessions;
3. severity of condition in participants with COPD (GOLD assessment[1] A and B versus C and D.);
4. sex; and
5. exercise intensity

Relevant subgroup analyses may be extended to random-effect meta-regression analyses if an adequate number of studies includes a specific outcome. For example, this could be subgroup analysis 1 with more than two categorical variables (i.e., no matching, subjectively measured PA, and objectively measured PA) or subgroup analysis 2 with the duration of intervention as a continuous explanatory variable. The outcome variable will be the effect estimate, i.e., MD or SMD.

Sensitivity analysis

We plan to carry out the following sensitivity analyses, removing the following from the primary outcome analyses:

- Trials judged in the quality assessment to be at high risk of bias (RoB 2), moderate or critical risk of bias (ROBINS-I) or poor quality (Downs and Black).

We will compare results from a fixed-effect model versus a random-effect model.

The sensitivity analyses will be presented in a summary table. Other suitable sensitivity analyses, as identified during the review process, may also be performed.

Assessment of the certainty of the evidence

For peer review only - http://bmjopen.bmj.com/site/about/guidelines.xhtml

We will use the Grading of Recommendations Assessment, Development, and Evaluation (GRADE) approach to assess the quality of a body of evidence related to the outcomes. Thus, the quality of the evidence will be graded as "high", "moderate", "low" or "very low". We will use the methods and recommendations described in the *Cochrane Handbook for Systematic Reviews of Interventions* Version 6.2[41] using GRADEpro software.

Patient and public involvement

This research is based on previously conducted studies and does not include new participants. Patients and/or the public were not involved in the design, conduct, reporting, or dissemination plans of this research.

DISCUSSION

The findings will increase our knowledge of whether and which responses and adaptations are altered, or even blunted, in people with COPD compared to healthy individuals without COPD. These findings will shed light on COPD as a systemic disease with extrapulmonary manifestations and how these manifestations impact the response to physical stress, such as exercise training. Consequently, exercise interventions could be better tailored to optimize pulmonary rehabilitation, physical capacity, and treatment in people with COPD.

Registration and publication of this protocol will promote and maintain transparency in the review process and minimize the risk of biases and counteract duplication of similar reviews. In addition, a high methodological standard will be supported by adhering to PRISMA-P, SWiM, and MECIR guidelines.

A potential limitation of this systematic review is the exclusion of literature written in languages not known by the research group might leave relevant literature out of the systematic review.

ETHICS AND DISSEMINATION

As this study is a systematic review, ethical approval is not required. The final review will be submitted for publication in a peer-reviewed journal. We also intend to present the findings at national and international conferences and inform patient organizations. There has been no patient or public involvement in the design of the protocol or dissemination plans.

Amendments

We will conduct the review according to this protocol and justify any deviations in a specific section of the systematic review.

Funding

This review is being undertaken as part of JJ's Ph.D. work, funded by the Swedish Research Council (Vetenskapsrådet), grant number: 2020-01296. The funder has no role in developing the protocol.

Competing interests

None declared.

Patient consent

For peer review only - http://bmjopen.bmj.com/site/about/guidelines.xhtml

Not required.

Author Contributions

AN conceptualized the study as the guarantor for the protocol. JJ and AN contributed to developing the eligibility criteria, the search strategy, the risk of bias assessment, data extraction, and data synthesis. JJ wrote the initial protocol, while AN and JDB provided critical insight and revisions to the manuscript. All authors read, approved, and contributed to the final written manuscript.

For peer review only

For peer review only - http://bmjopen.bmj.com/site/about/guidelines.xhtml

References

1. Global Initiative for Chronic Obstructive Lung Disease. Global Strategy for the Diagnosis, Management, and Prevention of Chronic Obstructive Pulmonary Disease 2022 Report 2022 [cited 2022 2022-06-14]. accessed 2022-06-14 2022.
2. Adeloye D, Chua S, Lee C, et al. Global and regional estimates of COPD prevalence: Systematic review and meta-analysis. *J Glob Health* 2015;5(2):020415. doi: 10.7189/jogh.05-020415 [published Online First: 2016/01/13]
3. World Health Organization. The top 10 causes of death World Health Organization; 2020 [cited 2021. Available from: https://www.who.int/news-room/fact-sheets/detail/the-top-10-causes-of-death accessed 2021-09-07 2021.
4. Lopez AD, Shibuya K, Rao C, et al. Chronic obstructive pulmonary disease: current burden and future projections. *Eur Respir J* 2006;27(2):397-412. doi: 10.1183/09031936.06.00025805 [published Online First: 2006/02/03]
5. Sleeman KE, de Brito M, Etkind S, et al. The escalating global burden of serious health-related suffering: projections to 2060 by world regions, age groups, and health conditions. *Lancet Glob Health* 2019;7(7):e883-e92. doi: https://doi.org/10.1016/S2214-109X(19)30172-X
6. McCarthy B, Casey D, Devane D, et al. Pulmonary rehabilitation for chronic obstructive pulmonary disease. *Cochrane Database Syst Rev* 2015(2):Cd003793. doi: 10.1002/14651858.CD003793.pub3 [published Online First: 2015/02/24]
7. Chatila WM, Thomashow BM, Minai OA, et al. Comorbidities in chronic obstructive pulmonary disease. *Proc Am Thorac Soc* 2008;5(4):549-55. doi: 10.1513/pats.200709-148ET [published Online First: 2008/05/06]
8. Putcha N, Drummond MB, Wise RA, et al. Comorbidities and Chronic Obstructive Pulmonary Disease: Prevalence, Influence on Outcomes, and Management. *Semin Respir Crit Care Med* 2015;36(4):575-91. doi: 10.1055/s-0035-1556063 [published Online First: 2015/08/03]
9. Smith MC, Wrobel JP. Epidemiology and clinical impact of major comorbidities in patients with COPD. *Int J Chron Obstruct Pulmon Dis* 2014;9:871-88. doi: 10.2147/COPD.S49621
10. Maltais F, Decramer M, Casaburi R, et al. An official American Thoracic Society/European Respiratory Society statement: update on limb muscle dysfunction in chronic obstructive pulmonary disease. *Am J Respir Crit Care Med* 2014;189(9):e15-62. doi: 10.1164/rccm.201402-0373ST [published Online First: 2014/05/03]
11. Agusti AG. COPD, a multicomponent disease: implications for management. *Respir Med* 2005;99(6):670-82. doi: 10.1016/j.rmed.2004.11.006 [published Online First: 2005/05/10]
12. Vanfleteren LE, Spruit MA, Groenen M, et al. Clusters of comorbidities based on validated objective measurements and systemic inflammation in patients with chronic obstructive pulmonary disease. *Am J Respir Crit Care Med* 2013;187(7):728-35. doi: 10.1164/rccm.201209-1665OC
13. Agusti A, Calverley PM, Celli B, et al. Characterisation of COPD heterogeneity in the ECLIPSE cohort. *Respir Res* 2010;11(1):122. doi: 10.1186/1465-9921-11-122 [published Online First: 20100910]
14. Emtner M, Wadell K. Effects of exercise training in patients with chronic obstructive pulmonary disease--a narrative review for FYSS (Swedish Physical Activity Exercise Prescription Book). *Br J Sports Med* 2016;50(6):368-71. doi: 10.1136/bjsports-2015-095872 [published Online First: 2016/01/30]
15. Chang SS, Chen S, McAvay GJ, et al. Effect of coexisting chronic obstructive pulmonary disease and cognitive impairment on health outcomes in older adults. *J Am Geriatr Soc* 2012;60(10):1839-46. doi: 10.1111/j.1532-5415.2012.04171.x [published Online First: 2012/10/06]
16. Mostert R, Goris A, Weling-Scheepers C, et al. Tissue depletion and health related quality of life in patients with chronic obstructive pulmonary disease. *RespirMed* 2000;94(9):859-67.

For peer review only - http://bmjopen.bmj.com/site/about/guidelines.xhtml

17. Gosselink R, Troosters T, Decramer M. Peripheral muscle weakness contributes to exercise limitation in COPD. *Am J Respir Crit Care Med* 1996;153:976-80.

18. Hamilton AL, Killian KJ, Summers E, et al. Muscle strength, symptom intensity and exercise capacity in patients with cardiorespiratory disorders. *Am J Respir Crit Care Med* 1995;152:2021-31.

19. Coronell C, Orozco-Levi M, Mendez R, et al. Relevance of assessing quadriceps endurance in patients with COPD. *Eur Respir J* 2004;24(1):129-36. [published Online First: 2004/08/06]

20. Decramer M, Gosselink R, Troosters T, et al. Muscle weakness is related to utilization of health care resources in COPD patients. *Eur Respir J* 1997;10(2):417-23. [published Online First: 1997/02/01]

21. Gosselink R, Troosters T, Decramer M. Distribution of muscle weakness in patients with stable chronic obstructive pulmonary disease. *J Cardiopulm Rehabil* 2000;20(6):353-60. doi: 10.1097/00008483-200011000-00004 [published Online First: 2001/01/06]

22. Marquis K, Debigaré R, Lacasse Y, et al. Midthigh muscle cross- sectional area is a better predictor of mortality than body mass index in patients with chronic obstructive pulmonary disease. *Am J Respir Crit Care Med* 2002;166(6):809.

23. Swallow EB, Gosker HR, Ward KA, et al. A novel technique for nonvolitional assessment of quadriceps muscle endurance in humans. *J Appl Physiol (1985)* 2007;103(3):739-46. doi: 10.1152/japplphysiol.00025.2007 [published Online First: 2007/06/16]

24. Pedersen BK, Saltin B. Exercise as medicine - evidence for prescribing exercise as therapy in 26 different chronic diseases. *Scand J Med Sci Sports* 2015;25 Suppl 3:1-72. doi: 10.1111/sms.12581 [published Online First: 2015/11/26]

25. Spruit MA, Burtin C, De Boever P, et al. COPD and exercise: does it make a difference? *Breathe (Sheffield, England)* 2016;12(2):e38-e49. doi: 10.1183/20734735.003916

26. Wang T, Mao L, Wang J, et al. Influencing Factors and Exercise Intervention of Cognitive Impairment in Elderly Patients with Chronic Obstructive Pulmonary Disease. *Clin Interv Aging* 2020;15:557-66. doi: 10.2147/CIA.S245147

27. Maltais F, LeBlanc P, Jobin J, et al. Intensity of training and physiologic adaptation in patients with chronic obstructive pulmonary disease. *Am J Respir Crit Care Med* 1997;155(2):555-61. doi: 10.1164/ajrccm.155.2.9032194 [published Online First: 1997/02/01]

28. Fermont JM, Masconi KL, Jensen MT, et al. Biomarkers and clinical outcomes in COPD: a systematic review and meta-analysis. *Thorax* 2019;74(5):439-46. doi: 10.1136/thoraxjnl-2018-211855 [published Online First: 2019/01/09]

29. Gloeckl R, Marinov B, Pitta F. Practical recommendations for exercise training in patients with COPD. *European Respiratory Review* 2013;22(128):178. doi: 10.1183/09059180.00000513

30. Latimer LE, Constantin-Teodosiu D, Popat B, et al. Whole-body & muscle responses to aerobic exercise training and withdrawal in ageing & COPD. *Eur Respir J* 2021 doi: 10.1183/13993003.01507-2021 [published Online First: 20210929]

31. Rabinovich RA, Ardite E, Troosters T, et al. Reduced muscle redox capacity after endurance training in patients with chronic obstructive pulmonary disease. *Am J Respir Crit Care Med* 2001;164(7):1114-8. doi: 10.1164/ajrccm.164.7.2103065

32. Puente-Maestu L, Tena T, Trascasa C, et al. Training improves muscle oxidative capacity and oxygenation recovery kinetics in patients with chronic obstructive pulmonary disease. *Eur J Appl Physiol* 2003;88(6):580-7. doi: 10.1007/s00421-002-0743-9 [published Online First: 20021130]

33. Costes F, Gosker H, Feasson L, et al. Impaired exercise training-induced muscle fiber hypertrophy and Akt/mTOR pathway activation in hypoxemic patients with COPD. *J Appl Physiol (1985)* 2015;118(8):1040-9. doi: 10.1152/japplphysiol.00557.2014 [published Online First: 20150219]

34. Gouzi F, Préfaut C, Abdellaoui A, et al. Blunted muscle angiogenic training-response in COPD patients versus sedentary controls. *Eur Respir J* 2013;41(4):806. doi: 10.1183/09031936.00053512

For peer review only - http://bmjopen.bmj.com/site/about/guidelines.xhtml

For peer review only

35. Castagna O, Boussuges A, Vallier JM, et al. Is impairment similar between arm and leg cranking exercise in COPD patients? *Respir Med* 2007;101(3):547-53. doi: https://doi.org/10.1016/j.rmed.2006.06.019
36. Franssen FME, Wouters EFM, Baarends EM, et al. Arm mechanical efficiency and arm exercise capacity are relatively preserved in chronic obstructive pulmonary disease. *Med Sci Sports Exerc* 2002;34(10)
37. Shamseer L, Moher D, Clarke M, et al. Preferred reporting items for systematic review and meta-analysis protocols (PRISMA-P) 2015: elaboration and explanation. *BMJ* 2015;350:g7647. doi: 10.1136/bmj.g7647 [published Online First: 2015/01/04]
38. Page MJ, McKenzie JE, Bossuyt PM, et al. The PRISMA 2020 statement: an updated guideline for reporting systematic reviews. *BMJ* 2021;372:n71. doi: 10.1136/bmj.n71
39. Spruit MA, Singh SJ, Garvey C, et al. An official American Thoracic Society/European Respiratory Society statement: key concepts and advances in pulmonary rehabilitation. *Am J Respir Crit Care Med* 2013;188(8):e13-64. doi: 10.1164/rccm.201309-1634ST [published Online First: 2013/10/17]
40. Nyberg A, Probst V, Vaes AW. Assessments. In: Holland AE, Corso SD, Spruit MA, eds. Pulmonary Rehabilitation: European Respiratory Society 2021:360.
41. Higgins JPT TJ, Chandler J, Cumpston M, Li T, Page MJ, Welch VA (editors). Cochrane Handbook for Systematic Reviews of Interventions version 6.2 (updated February 2021): Cochrane, 2021, 2021.
42. Sterne JA, Savović J, Page MJ, et al. RoB 2: a revised tool for assessing risk of bias in randomised trials. *BMJ* 2019;366
43. Sterne JA, Hernán MA, Reeves BC, et al. ROBINS-I: a tool for assessing risk of bias in non-randomised studies of interventions. *BMJ* 2016;355
44. Downs SH, Black N. The feasibility of creating a checklist for the assessment of the methodological quality both of randomised and non-randomised studies of health care interventions. *J Epidemiol Community Health* 1998;52(6):377-84. doi: 10.1136/jech.52.6.377
45. Deeks JJ, Dinnes J, D'Amico R, et al. Evaluating non-randomised intervention studies. *Health Technol Assess* 2003;7(27):iii-x, 1-173. doi: 10.3310/hta7270
46. Frykholm E, Lima VP, Selander HV, et al. Physiological and Symptomatic Responses to Arm versus Leg Activities in People with Chronic Obstructive Pulmonary Disease: A Systematic Review and Meta-Analysis. *Copd* 2019;16(5-6):390-405. doi: 10.1080/15412555.2019.1674269 [published Online First: 20191021]
47. Campbell M, McKenzie JE, Sowden A, et al. Synthesis without meta-analysis (SWiM) in systematic reviews: reporting guideline. *BMJ* 2020;368:l6890. doi: 10.1136/bmj.l6890

For peer review only

For peer review only - http://bmjopen.bmj.com/site/about/guidelines.xhtml

Reporting checklist for protocol of a systematic review and meta analysis.

Moher D, Shamseer L, Clarke M, Ghersi D, Liberati A, Petticrew M, Shekelle P, Stewart LA. Preferred Reporting Items for Systematic Review and Meta-Analysis Protocols (PRISMA-P) 2015 statement. Syst Rev. 2015;4(1):1.

		Reporting Item	Page
**Title**			
Identification	#1a	Identify the report as a protocol of a systematic review	1
Update	#1b	If the protocol is for an update of a previous systematic review, identify as such	-
**Registration**			
	#2	If registered, provide the name of the registry (such as PROSPERO) and registration number	2
**Authors**			
Contact	#3a	Provide name, institutional affiliation, e-mail address of all protocol authors; provide physical mailing address of corresponding author	1
Contribution	#3b	Describe contributions of protocol authors and identify the guarantor of the review	14
**Amendments**			
	#4	If the protocol represents an amendment of a previously completed or published protocol, identify as such and list changes; otherwise, state plan for documenting important protocol amendments	13
**-Support**			
Sources	#5a	Indicate sources of financial or other support for the review	13
Sponsor	#5b	Provide name for the review funder and / or sponsor	n/a
Role of sponsor or funder	#5c	Describe roles of funder(s), sponsor(s), and / or institution(s), if any, in developing the protocol	13
**Introduction**			
Rationale	#6	Describe the rationale for the review in the context of what is already known	5
Objectives	#7	Provide an explicit statement of the question(s) the review will address with reference to participants, interventions, comparators, and outcomes (PICO)	5 (6)
**Methods**			
Eligibility criteria	#8	Specify the study characteristics (such as PICO, study design, setting, time frame) and report characteristics (such as years considered, language,	5-7

For peer review only

		publication status) to be used as criteria for eligibility for the review	
Information sources	#9	Describe all intended information sources (such as electronic databases, contact with study authors, trial registers or other grey literature sources) with planned dates of coverage	7
Search strategy	#10	Present draft of search strategy to be used for at least one electronic database, including planned limits, such that it could be repeated	8 + appendix
Study records - data management	#11a	Describe the mechanism(s) that will be used to manage records and data throughout the review	8-9
Study records - selection process	#11b	State the process that will be used for selecting studies (such as two independent reviewers) through each phase of the review (that is, screening, eligibility and inclusion in meta-analysis)	8-9
Study records - data collection process	#11c	Describe planned method of extracting data from reports (such as piloting forms, done independently, in duplicate), any processes for obtaining and confirming data from investigators	8-9
Data items	#12	List and define all variables for which data will be sought (such as PICO items, funding sources), any pre-planned data assumptions and simplifications	9
Outcomes and prioritization	#13	List and define all outcomes for which data will be sought, including prioritization of main and additional outcomes, with rationale	9
Risk of bias in individual studies	#14	Describe anticipated methods for assessing risk of bias of individual studies, including whether this will be done at the outcome or study level, or both; state how this information will be used in data synthesis	9-11
Data synthesis	#15a	Describe criteria under which study data will be quantitatively synthesised	11-12
Data synthesis	#15b	If data are appropriate for quantitative synthesis, describe planned summary measures, methods of handling data and methods of combining data from studies, including any planned exploration of consistency (such as I2, Kendall's τ)	12
Data synthesis	#15c	Describe any proposed additional analyses (such as sensitivity or subgroup analyses, meta-regression)	12
Data synthesis	#15d	If quantitative synthesis is not appropriate, describe the type of summary planned	11
Meta-bias(es)	#16	Specify any planned assessment of meta-bias(es) (such as publication bias across studies, selective reporting within studies)	12
Confidence in cumulative evidence	#17	Describe how the strength of the body of evidence will be assessed (such as GRADE)	13

None The PRISMA-P elaboration and explanation paper is distributed under the terms of the Creative Commons Attribution License CC-BY. This checklist can be completed online using https://www.goodreports.org/, a tool made by the EQUATOR Network in collaboration with Penelope.ai

For peer review only

Database:
Ovid MEDLINE(R) and Epub Ahead of Print, In-Process, In-Data-Review & Other Non-Indexed Citations, Daily and Versions(R) <1946 to February 15, 2022>

Search as per 2022-02-15:

1. exp Pulmonary Disease, Chronic Obstructive/ (62214)
2. COPD.mp. (53231)
3. Chronic Obstructive Pulmonary Disease.mp. (55582)
4. Chronic Obstructive Airway Disease.mp. (309)
5. Chronic Obstructive Lung Disease.mp. (4419)
6. Airflow Obstruction$, Chronic.mp. (11)
7. Airflow limitation.mp. (3405)
8. exp Exercise/ (226020)
9. exp Exercise Therapy/ (58389)
10. Exercise Program.mp. (9485)
11. High-Intensity Interval.mp. (3443)
12. exp High-Intensity Interval Training/ (1691)
13. Interval Exercise.mp. (862)
14. Intermittent Exercise.mp. (1174)
15. Intermittent Training.mp. (191)
16. Continuous Exercise.mp. (788)
17. Continuous Training.mp. (1403)
18. Moderate-Intensity Continuous Exercise.mp. (173)
19. Sprint interval training.mp. (384)
20. Physical Exercise.mp. (18246)
21. Pulmonary Rehab*.mp. (4422)
22. Aerobic Exercise.mp. (11132)
23. Anaerobic exercise.mp. (622)
24. Exercise Training.mp. (19075)
25. exp Endurance Training/ (464)
26. Strength Training.mp. (5996)
27. exp Resistance Training/ (10819)
28. NMES.mp. (1389)
29. Neuromuscular Stimulation.mp. (525)
30. Inspiratory Muscle Training.mp. (831)
31. Cycling.mp. (70584)
32. Tai Chi.mp. (2083)
33. exp Tai Ji/ (1305)
34. exp Walking/ (62002)
35. 1 or 2 or 3 or 4 or 5 or 6 or 7 (97573)
36. 8 or 9 or 10 or 11 or 12 or 13 or 14 or 15 or 16 or 17 or 18 or 19 or 20 or 21 or 22 or 23 or 24 or 25 or 26 or 27 or 28 or 29 or 30 or 31 or 32 or 33 or 34 (374060)
37. 35 and 36 (6309)
38. exp animal/ not humans.sh. (4959724)
39. 37 not 38 (6264)

For peer review only

BMJ Open

Physiological Responses and Adaptations to Exercise Training in People with or without Chronic Obstructive Pulmonary Disease: Protocol for a Systematic Review and Meta-Analysis

Journal:	*BMJ Open*
Manuscript ID	bmjopen-2022-065832.R1
Article Type:	Protocol
Date Submitted by the Author:	29-Aug-2022
Complete List of Authors:	Jakobsson, Johan; Umea University, Community Medicine and Rehabilitation, Physiotherapy<br>De Brandt, Jana; Umea University, Community Medicine and Rehabilitation, Physiotherapy<br>Nyberg, André; Umea University, Department of Community Medicine and Rehabilitation, Physiotherapy
**Primary Subject Heading**:	Sports and exercise medicine
Secondary Subject Heading:	Respiratory medicine, Rehabilitation medicine, Public health
Keywords:	Respiratory physiology < THORACIC MEDICINE, Physiology < NATURAL SCIENCE DISCIPLINES, SPORTS MEDICINE, Chronic airways disease < THORACIC MEDICINE

SCHOLARONE™ Manuscripts

Physiological Responses and Adaptations to Exercise Training in People with or without Chronic Obstructive Pulmonary Disease: Protocol for a Systematic Review and Meta-Analysis

Jakobsson, J*[1]., De Brandt, J[1]., Nyberg, A[1].

***Corresponding author:** Johan Jakobsson, johan.jakobsson@umu.se, +46 70 271 46 01

Author affiliations

1 Section of Physiotherapy, Department of Community Medicine and Rehabilitation, Faculty of Medicine, Umeå University, Linneaus Väg 8, 907 36 Umeå

Johan Jakobsson: johan.jakobsson@umu.se

Jana De Brandt: jana.de.brandt@umu.se

Andre Nyberg: andre.nyberg@umu.se

Word count: 3933 (Background to Amendments)

Keywords: COPD; endurance training; resistance training; pulmonary rehabilitation; acute exercise

For peer review only

ABSTRACT

Introduction

Exercise training is a cornerstone in managing chronic obstructive pulmonary disease (COPD), leading to several physiological adaptations including, but not limited to, structural and muscular alterations, increased exercise capacity, and improved cognitive function. Still, it is not uncommon that the acute physiological response to an exercise session and the extent of chronic adaptations to exercise training are altered compared to people without COPD. To date, potential differences in acute physiological responses and chronic adaptations in people with or without COPD are not fully understood, and results from individual studies are contradictory. Therefore, the current study aims to synthesize and compare the acute physiological responses and chronic adaptations to exercise training in people with COPD compared to people without COPD.

Methods and analyses

A systematic review of randomised controlled trials (RCT), non-randomized studies of interventions (NRSI), and cross-sectional studies (CSS) will be conducted. A comprehensive search strategy will identify relevant studies from MEDLINE, Scopus, CINAHL, SPORTDiscus, CENTRAL, and Cochrane Airways Trials Register databases. Studies including adults with and without COPD will be considered. Outcomes will include cardiorespiratory, muscular, and cognitive function, intramuscular adaptations, lung volumes, and cardiometabolic responses. The protocol is reported according to the Preferred Reporting Items for Systematic Reviews and Meta-Analyses Protocols and the Cochrane Methodological Expectations of Cochrane Intervention Reviews. Risk of bias assessment will be conducted using Cochranes Risk-of-Bias 2 tool (for RCTs), Risk-of-Bias in Non-Randomized Studies Tool (for NRSIs), and Downs and Black checklist (for CCS). Meta-analyses will be conducted when appropriate, supplemented with a systematic synthesis without meta-analysis.

Ethics and dissemination

As this study is a systematic review, ethical approval is not required. The final review results will be submitted for publication in a peer-reviewed journal and presented at international conferences.

PROSPERO registration number: CRD42022307577

For peer review only - http://bmjopen.bmj.com/site/about/guidelines.xhtml

Article Summary

Strengths and limitations of this study

- This protocol is reported according to the Preferred Reporting Items for Systematic Reviews and Meta-Analyses Protocols.
- The protocol includes a comprehensive and peer-reviewed search strategy and broad inclusion criteria to comprehensively synthesise available evidence.
- Exclusion of literature written in languages not known by the research group might exclude relevant literature from the systematic review.

For peer review only

For peer review only - http://bmjopen.bmj.com/site/about/guidelines.xhtml

BACKGROUND

Chronic obstructive pulmonary disease (COPD) is a chronic respiratory disease characterized by persistent and progressive airflow limitation, causing breathlessness, productive coughing, fatigue, and recurrent chest infection.[1] COPD is highly prevalent, with the global prevalence among individuals aged ≥40 years being 11.7%.[2] Worldwide, COPD was the third leading cause of death in 2019, according to the World Health Organization,[3] which is expected to increase during the next four decades.[4 5]

Although COPD is primarily a respiratory disease, it is best understood as a systemic disease with several extrapulmonary manifestations.[6 7] Most people (40-98%[8-10]) with COPD have comorbidities such as cardiovascular disease, diabetes or metabolic syndrome, muscle atrophy, cognitive dysfunction, or muscle dysfunction[6 11 12] that directly and substantially impact the disease. On average, a person with COPD suffers from four extrapulmonary manifestations[13], observed across the entire spectrum of airflow limitation severity.[14] Having one or more comorbidities is associated with more hospitalizations and increased mortality.[15] Nevertheless, extrapulmonary manifestations are an overlooked aspect of COPD that is not dealt with optimally even though they negatively impact important clinical outcomes independent of the degree of lung impairment.[11 16] For instance, there is an intimate connection between reductions in limb muscle strength and endurance capacity with a reduced quality of life,[17] exercise intolerance,[18-20] greater healthcare utilization,[21] decreased ability to perform daily activities,[22] and increased mortality.[23 24] For example, quadriceps muscle atrophy is associated with a 4-fold increase in mortality even after adjusting for age, sex, and lung function.[11] At the same time, people with coexisting COPD and cognitive dysfunction have a mortality rate nearly 3-times as high compared to those having each condition alone.[16]

Similar to the healthy population and most chronic diseases,[25] exercise training is one of the cornerstones in treating extrapulmonary manifestations in COPD, such as decreased muscle, cardiorespiratory or cognitive function.[1 15 26 27] Regular exercise training in people with COPD can increase cardiorespiratory fitness, partly due to increased mitochondrial density and oxidative enzyme activity.[28] Importantly, exercise training has also been shown to reduce dyspnea and fatigue during daily life activities, decrease anxiety and depression, and improve health-related quality of life.[29] It is evident that exercise training improves multiple extrapulmonary manifestations in people with COPD. However, the acute physiological response and the extent of chronic adaptations are repeatedly reported to be altered compared to healthy individuals without COPD and vary amongst people with COPD[30]. Recently, it was shown that the response to aerobic training concerning mitochondrial function is blunted in COPD compared to matched individuals without COPD.[31] However, a blunted response to exercise training is not a universal finding. For instance, Rabinovich et al. [32] reported a difference in oxidative stress between people with COPD and those without COPD following high-intensity training, while Puente-Maestu[33] did not. Costes et al.,[34] found no change in capillary-to-fiber ratio and mean fiber size in people with COPD after multidisciplinary exercise training, while Gouzi et al.[35] showed similar increases in those with and without COPD following endurance training. One study showed that people with COPD have lower mechanical efficiency and exercise capacity in the upper limbs than those without COPD,[36]

For peer review only - http://bmjopen.bmj.com/site/about/guidelines.xhtml

while others reported preserved capacities.[37] Thus, although multiple studies have been conducted, these seemingly contradictory findings highlight the need for a systematic comparison of studies evaluating acute responses and chronic adaptations to exercise training in people with COPD compared to those without COPD. Increased knowledge about altered and even blunted acute and chronic responses to exercise training in COPD is needed to better tailor exercise training in people with COPD.

Aims and objectives

This systematic review and meta-analysis aims to synthesize the acute physiological responses and chronic adaptations to exercise training in people with COPD compared to people without COPD. Specifically, the systematic review will address the question: Are there differences in acute physiological responses and chronic adaptations to exercise training in people with or without COPD?

The primary objective is to compare the chronic physiological adaptations to exercise training in people with or without COPD performing the same exercise intervention. The secondary objective is to compare the acute physiological response to exercise training in people with or without COPD performing the same exercise session.

METHODS

The study protocol of this systematic review is reported in line with the Preferred Reporting Items for Systematic Reviews and Meta-Analyses Protocols (PRISMA-P) guideline (see supplemental appendix 1)[38] and Cochrane Methodological Expectations of Cochrane Intervention Reviews (MECIR). The systematic review will be reported according to PRISMA 2020 guidelines[39], and was registered in the International Prospective Register of Systematic Reviews (PROSPERO) on March 27, 2022 (CRD42022307577).

Eligibility criteria

Studies will be selected according to the criteria outlined below.

Types of study designs

Randomized controlled trials (RCTs), controlled non-randomized studies of interventions (NRSI), cluster trials and prospective comparative cohort studies will be included to synthesize chronic physiological adaptations to exercise training in people with COPD in comparison to people without COPD. Studies presenting a cross-sectional comparison of acute physiological responses to an exercise training session will be included to compare acute physiological responses between people with and without COPD. Retrospective studies, case reports, case series, and opinion pieces will be excluded.

Types of participants

Any study including adults ≥18 years of age with a diagnosis of COPD and a group of individuals without COPD (often referred to as "healthy controls") will be considered. There will be no eligibility criteria for sex or ethnicity or comorbidities in the group of people without COPD. Participants with COPD and comorbidities will not be excluded, but COPD should be the primary disease.

For peer review only - http://bmjopen.bmj.com/site/about/guidelines.xhtml

Types of intervention

Any type of acute exercise training session and exercise training interventions will be considered as long as data can be separated for and compared between people with COPD and those without COPD. For example, but not limited to endurance training, interval training, resistance/strength training, neuromuscular electrical stimulation (NMES), or inspiratory muscle training (IMT). For chronic adaptations to exercise training, interventions with a duration of fewer than three weeks will be excluded.

Types of comparator

This systematic review will investigate the differential effects of the same intervention given to different populations, as such, there is no comparator/control group.

Types of outcome measures

Studies will not be excluded based on outcomes. The following outcomes are of interest, including but not limited to:

- Cardiorespiratory function
  - Maximal exercise performance: peak oxygen consumption (VO_2peak) and maximal aerobic power during an incremental exercise test.
  - Exercise tolerance: constant-work rate cycle test, endurance shuttle walk test, step tests
- Cardiorespiratory and metabolic responses during exercise: e.g. heart rate, peripheral capillary oxygen saturation, oxygen consumption, carbon dioxide production, respiratory exchange ratio, the concentration of blood lactate, markers of mitochondrial biogenesis, myogenesis, neuroprotection, inflammation, and oxidative stress.
- Muscle function:
  - Muscle strength: Maximal muscle force (e.g., maximal voluntary contraction), isokinetic or isometric (peak torque), and isotonic strength (1-repetition maximum).
  - Muscle power: Repeated maximal contractions at a submaximal load (i.e., to generate an increase in contractile force over a certain unit of time).
  - Muscle endurance: Change in muscle force over time (e.g., time to failure when maximal or submaximal static or dynamic contractions are performed).
- Functional performance: e.g. 6-minute-walk-test, 5-time sit-to-stand test, 30-sec sit-to-stand and 1-minute sit-to-stand test.
- Intramuscular adaptations: e.g. Mitochondrial function and markers, markers of oxidative stress and inflammation, enzyme activity, fibre type proportion, fibre size, and capillarization.
- Lung volumes: e.g. Tidal volume, minute ventilation, breathing frequency, ventilation to maximum voluntary ventilation ratio, dynamic hyperinflation, end-expiratory lung volume, end-inspiratory lung volume, and inspiratory capacity.
- Cognitive function: Global cognitive tests (e.g. Montreal Cognitive Assessment, Mini-Mental State Examination, General Practitioner Assessment of Cognition, Mini-Cog Test).

Timing

For peer review only

Only before and immediately after intervention data will be included for intervention studies with more than two time points of assessments. If an intervention study measures acute responses before and after the intervention, only before (baseline) measurements will be included.

Setting

There will be no exclusion by type of setting.

Timespan

All articles from inception to the start of the literature search will be considered. Any restriction of the time period is not deemed relevant.

Language

English, Swedish, Norwegian, Danish, French, and Dutch literature will be considered. Articles written in other languages will be excluded.

SEARCH METHODS FOR IDENTIFICATION OF STUDIES

Electronic searches

Relevant studies will be identified from (database inception to date):

- MEDLINE (Ovid SP interface)
- Scopus
- CINAHL and SPORTDiscus (EBSCO interface)
- Cochrane Central Register of Controlled Trials (CENTRAL)
- Cochrane Airways Trials Register

Search for grey literature, registered trials, and ongoing studies will be performed, from the inception of the database to date, through but not limited to:

- World Health Organization International Clinical Trials Registry Platform (ICTRP)
- International Standard Randomised Controlled Trial Number (ISRCTN)
- National Institutes of Health Clinical Trials Database (ClinicalTrials.gov)

Corresponding authors or principal investigators of identified unpublished and studies in progress will be contacted to establish whether published literature was missed.

Other resources

Relevant conference proceedings will be searched, and if required, we will contact authors of identified trials for unpublished studies or missing information. If sufficient data is provided, conference abstracts will be included.

The reference list of included studies identified through the search will be hand-searched for additional studies. Other systematic reviews on the topic will also be checked for further studies. The 'related articles' function in PubMed will be used on included studies or relevant reviews.

The Cochrane Database of Systematic Reviews and PROSPERO have also been searched for existing or ongoing reviews on the topic. No previous or ongoing systematic review was

For peer review only - http://bmjopen.bmj.com/site/about/guidelines.xhtml

For peer review only

identified. We will search PubMed for errata or retractions from included studies published in full text and report which date this was done within the review.

Search strategy

The search strategy is based on the Cochrane Airways Group Register of Trials guidelines. It has been developed with the assistance of a health science librarian and is peer-reviewed by experts in exercise training, pulmonary rehabilitation, and lung diseases. To ensure literature saturation, comprehensive searches will be constructed of both index terming (MeSH terms), free text terms, and synonyms. No language restriction will be imposed on the search. A draft of the search strategy for MEDLINE (OVID interface) is presented in the online supplemental appendix 2. The final MEDLINE search strategy will be adapted to other databases' and trial registries' syntax and subject headings.

Data collection and analysis

Study records

The results from the literature searches will be uploaded to Covidence (Covidence, Melbourne, Australia). This internet-based software program facilitates the management of studies, including removing duplicates and collaboration among reviewers during the study-selection process. The research team will develop, test, and refine screening questions and forms for assessments based on the eligibility criteria. Members of the review team not familiar with Covidence will be trained in the software before the start of the review. The search process will be documented, including:

- the name of the database searched
- the name of the database provider/system used
- the date when the search was run
- the years covered by the search
- the search terms used, hits per search term, and number of articles retrieved

Selection process

Two review authors (JJ and JDB) will screen the titles and abstracts of the search results independently and code them as 'retrieve' (eligible or potentially eligible/unclear) or 'do not retrieve'. Then, we will retrieve the full-text study reports of all potentially eligible studies. Two review authors (JJ and JDB) will independently screen them for inclusion, recording the reasons for exclusion of ineligible studies. If needed, additional information from study authors will be sought using e-mail to resolve questions about eligibility. We will resolve any disagreement through discussion or if required, we will consult a third review author (AN), and a majority rule will be used. Neither of the review authors will be blind to the journal titles, the study authors, or institutions. Special attention will be given to identifying and excluding duplicates and collating multiple reports of the same study. Rather than each report, each study is the unit of interest in the review. We will record the selection process in sufficient detail to complete a PRISMA flowchart diagram. The inter-assessor agreement will be assessed using Kappa statistics.

Data collection process

For peer review only

For peer review only - http://bmjopen.bmj.com/site/about/guidelines.xhtml

Two reviewers (JJ and JDB) will use a standardized data extraction form to extract data independently from full the texts of all included studies. The form will be pilot-tested on two to three potentially eligible articles and reviewers trained in using the form. Disagreement will be solved by consensus. When disagreement cannot be resolved by consensus, a third reviewer (AN) will be consulted, and a majority rule will be used. In the absence of complete descriptions of outcomes and effect estimates, authors will be asked for additional information or missing data using e-mail. All data will be double-checked with the included studies by a third reviewer (AN)**.** The following study characteristics will be extracted:

- Study: author(s) name, title, publication year
- Methods: study design, total duration of the study, number of study centers (if multicenter), study setting
- Participants: sample size, mean age, age range, sex, inclusion and exclusion criteria, baseline lung function, and the number of comorbidities. For COPD group: severity of the condition.
- Intervention: type of exercise training, intensity, duration, frequency, and progression principles
- Outcomes: Data will be sought on the following outcomes: Maximal exercise performance, exercise capacity, cardiorespiratory and metabolic responses during exercise, muscle strength, muscle power, muscle endurance, functional performance, intramuscular adaptations, and lung volumes. Chronic adaptation following an exercise intervention period in maximal exercise performance, exercise tolerance, and muscle function are the primary outcomes in this systematic review since these are vital objectives in international COPD treatment guidelines [6 40 41]. Other chronic adaptations and acute responses from a single exercise session in the abovementioned outcomes are secondary outcomes. The "Type of outcome measures" section includes detailed descriptions of selected outcomes.

One review author (JJ) will transfer data into Review Manager software (Revman, Cochrane Collaboration, Oxford, UK). We will double-check that data are entered correctly by comparing the data presented in the systematic review with the study reports. A second review author (JDB) will spot-check study characteristics for accuracy against the study report.

Risk of bias assessment

The risk of bias (RoB) in eligible studies will be assessed independently by two authors (JJ and JDB) using appropriate tools. We will resolve any disagreements by discussing or involving another author (AN). No study will be excluded due to poor quality, but sensitivity analyses excluding poor-quality studies will be performed.

RCTs will be assessed using Risk of Bias Tool 2 (RoB 2)[42 43]. We will assess the risk of bias in the following five domains:

1. bias arising from the randomisation process;
2. bias due to deviations from intended interventions;

For peer review only - http://bmjopen.bmj.com/site/about/guidelines.xhtml

3. bias due to missing outcome data;
4. bias in measurement of the outcome; and
5. bias in selection of the reported result.

Signalling questions and the algorithm in the RoB 2 tool will provide the basis for the judgment about the risk of bias. If there is sufficient reason to override the algorithm, we will do this and state a reason for it. To manage the assessment of bias of included studies we will use templates and spreadsheets of the RoB 2 tool.[43] An overall risk of bias for each result will be produced, based on the least favourable assessment of bias across the domains according to:

- low risk of bias;
- some concerns; or
- high risk of bias.

The Risk of Bias in Non-randomized Studies of Interventions (ROBINS-I)[44] will be used for assessing the quality of NRSIs. This is the recommended tool by the Cochrane Scientific Committee to assess the risk of bias in NRSIs. The ROBINS-I tool is similar to the RoB 2 tool and comprises a series of signalling questions assessing the bias in seven domains:

1. bias due to confounding;
2. bias in selection of participants for the study;
3. bias in classification of interventions;
4. bias due to deviations from intended interventions;
5. bias due to missing data;
6. bias in measurement of outcomes; and
7. bias in selection of the reported result

Important confounding domains that are prognostic factors for the outcomes of interest are the extent of matching between the groups with and without COPD, especially regarding daily physical activity (PA) and any differences in cardiorespiratory fitness.

We will reach a risk of bias judgment and assign one of the five levels to each domain and an overall judgment per study:

- low risk of bias;
- moderate risk of bias;
- serious risk of bias;
- critical risk of bias; or
- no information (on which to base a judgment about risk of bias)

For cross-sectional studies, the risk of bias will be assessed using a modified version of the checklist by Downs and Black[45]. This checklist has been recommended for assessing the quality of RCT and NRSI, as well as cross-sectional studies.[46] The 14 most relevant items will be used. The 14 items are appropriate for NRSI and have previously been used in studies within the field.[47] The psychometric properties of the checklist have been validated and reported

For peer review only - http://bmjopen.bmj.com/site/about/guidelines.xhtml

elsewere[45]. The 14 items, previously described[47] are yes (1 point) or no (0 points) questions resulting in a score between 0 and 14 where higher scores indicate higher methodological quality. For subsequent sensitivity analysis, the following cut-points, based on previously used cut-offs for the original list[48], will be used to categorize studies by quality: "Excellent" (13-14 points), "good" (10-12 points), "fair" (8-9 points) or "poor" ≤7 points). The 14 items checklist can be found in supplemental appendix 3:

Synthesis

Data will be meta-analysed in RevMan and will be presented in forest plots and a table providing a summary of study characteristics and the findings. Other computer software, such as Jamovi might also be used for supplemental analyses. For data that cannot be meta-analysed, i.e., due to a lack of data to calculate effect sizes or too diverse study characteristics, a systematic synthesis without meta-analysis (SWiM)[49] will be presented in the text and with graphics. The SWiM will be conducted according to the PRISMA and SWiM[49] reporting guidelines. The SWiM guideline is a nine-item checklist to promote transparent reporting of systematic reviews.[49]

Meta-analyses will be undertaken where meaningful; if the participants, type of intervention (i.e. resistance training, continuous training, high-intensity interval training), and study design (i.e. acute or chronic) are homogenous enough to warrant pooling. We anticipate only continuous data to be reported for the outcomes of interest. Continuous data will be analysed as the mean difference (MD) where outcome data are reported on a uniform scale or standardized mean difference (SMD) when different metrics are used but deemed clinically homogenous. Meta-analyses will be based on change from baseline rather than post-intervention values when available. Data extraction (and any needed imputations and calculations) will be done according to recommendations in *Cochrane Handbook for Systematic Reviews of Interventions* Version 6.2, section 6.5.2.[42] If needed, additional information from the study authors will be sought using e-mail. A random-effects model will be used to allow for random error and inter-study variability.

To compare the physiological effects of exercise, we will use per-protocol analyses as the effect of interest, where they are reported. If only intention-to-treat data is reported, this will be used for analysis. If both per-protocol and intention-to-treat data are reported, the intention-to-treat data will be used in a sensitivity analysis. Where multiple trial arms are reported in a single study, we will include only relevant ones. If applicable, we will combine intervention arms or reported subgroups as per the recommendations in *Cochrane Handbook for Systematic Reviews of Interventions* Version 6.2[42], sections 6.2 and 6.5, respectively.

Assessment of heterogeneity

We will use the I^2 and τ^2 statistics to measure heterogeneity between the studies in each analysis. If we identify substantial heterogeneity ($I^2 \geq 50\%$) we will report it and explore the possible causes by prespecified subgroup analysis. The importance of the I^2 statistic will be interpreted together with the confidence interval and P value from the chi-squared statistic.

Assessment of reporting biases

For peer review only - http://bmjopen.bmj.com/site/about/guidelines.xhtml

If we can pool more than ten studies we will create and examine the asymmetry of a funnel plot and perform Egger's test to explore possible small study and publication biases. The possible influence of small-study/publication biases on review findings will be a part of our 'Risk of bias' assessment and GRADE assessments of the quality of the evidence.

Subgroup analysis and investigation of heterogeneity

We plan to carry out the following subgroup analyses if adequate data can be obtained:

1. the extent of matching on PA (matched with any type of method compared to not matched);
2. duration of intervention ≥ 15 sessions compared to ≤ 16 sessions;
3. severity of the condition in participants with COPD (GOLD assessment[1] A and B versus C and D.);
4. sex;
5. exercise intensity; and
6. body mass index ≥ 21 compared to < 21 (a cut-off value often used in COPD[50] for underweight)

Relevant subgroup analyses may be extended to random-effect meta-regression analyses if an adequate number of studies includes a specific outcome. For example, this could be subgroup analysis 1 with more than two categorical variables (i.e., no matching, subjectively measured PA, and objectively measured PA) or subgroup analysis 2 with the duration of intervention as a continuous explanatory variable. The outcome variable will be the effect estimate, i.e., MD or SMD.

Sensitivity analysis

We plan to carry out the following sensitivity analyses, removing the following from the primary outcome analyses:

- Trials judged in the quality assessment to be at high risk of bias (RoB 2), moderate or critical risk of bias (ROBINS-I) or poor quality (Downs and Black).
- Per-protocol analyses (either removing the whole trial or using data for intention-to-treat if this is reported too)

We will compare results from a fixed-effect model versus a random-effect model. If we impute standard deviations using correlation coefficients in the synthesis, sensitivity analyses will be performed with different values of the correlation coefficient to determine if the overall results are robust to the imputed correlation coefficients[42]. When appropriate, we will compare the results from an absolute change from baseline versus a percentage change from baseline meta-analysis.

The sensitivity analyses will be presented in a summary table. Other suitable sensitivity analyses, as identified during the review process, may also be performed.

For peer review only - http://bmjopen.bmj.com/site/about/guidelines.xhtml

Assessment of the certainty of the evidence

We will use the Grading of Recommendations Assessment, Development, and Evaluation (GRADE) approach to assess the quality of a body of evidence related to the outcomes. Thus, the quality of the evidence will be graded as "high", "moderate", "low" or "very low". We will use the methods and recommendations described in the *Cochrane Handbook for Systematic Reviews of Interventions* Version 6.2[42] using GRADEpro software.

Patient and public involvement

Patients and/or the public were not involved in the design, conduct, reporting, or dissemination plans of this research.

DISCUSSION

Registration and publication of this protocol will promote and maintain transparency in the review process and minimize the risk of biases and counteract duplication of similar reviews. In addition, a high methodological standard will be supported by adhering to PRISMA-P, SWiM, and MECIR guidelines. A potential limitation of this systematic review is the exclusion of literature written in languages not known by the research group might leave relevant literature out of the systematic review.

ETHICS AND DISSEMINATION

As this study is a systematic review, ethical approval is not required. The systematic review will be submitted for publication in a peer-reviewed journal. We also intend to present the findings at national and international conferences and inform patient organizations. There has been no patient or public involvement in the design of the protocol or dissemination plans.

Amendments

We will conduct the review according to this protocol and justify any deviations in a specific section of the systematic review.

Funding

This review is being undertaken as part of JJ's Ph.D. work, funded by the Swedish Research Council (Vetenskapsrådet), grant number: 2020-01296. The funder has no role in developing the protocol.

Competing interests

None declared.

Patient consent

Not required.

Author Contributions

AN conceptualized the study as the guarantor for the protocol. JJ and AN contributed to developing the eligibility criteria, the search strategy, the risk of bias assessment, data extraction, and data synthesis. JJ wrote the initial protocol, while AN and JDB provided critical

For peer review only - http://bmjopen.bmj.com/site/about/guidelines.xhtml

insight and revisions to the manuscript. All authors read, approved, and contributed to the final written manuscript.

For peer review only

For peer review only - http://bmjopen.bmj.com/site/about/guidelines.xhtml

References

1. Global Initiative for Chronic Obstructive Lung Disease. Global Strategy for the Diagnosis, Management, and Prevention of Chronic Obstructive Pulmonary Disease 2022 Report 2022 [cited 2022 2022-06-14]. accessed 2022-06-14 2022.
2. Adeloye D, Chua S, Lee C, et al. Global and regional estimates of COPD prevalence: Systematic review and meta-analysis. *J Glob Health* 2015;5(2):020415. doi: 10.7189/jogh.05-020415 [published Online First: 2016/01/13]
3. World Health Organization. The top 10 causes of death World Health Organization; 2020 [cited 2021. Available from: https://www.who.int/news-room/fact-sheets/detail/the-top-10-causes-of-death accessed 2021-09-07 2021.
4. Lopez AD, Shibuya K, Rao C, et al. Chronic obstructive pulmonary disease: current burden and future projections. *Eur Respir J* 2006;27(2):397-412. doi: 10.1183/09031936.06.00025805 [published Online First: 2006/02/03]
5. Sleeman KE, de Brito M, Etkind S, et al. The escalating global burden of serious health-related suffering: projections to 2060 by world regions, age groups, and health conditions. *Lancet Glob Health* 2019;7(7):e883-e92. doi: https://doi.org/10.1016/S2214-109X(19)30172-X
6. McCarthy B, Casey D, Devane D, et al. Pulmonary rehabilitation for chronic obstructive pulmonary disease. *Cochrane Database Syst Rev* 2015(2):Cd003793. doi: 10.1002/14651858.CD003793.pub3 [published Online First: 2015/02/24]
7. Machado A, Marques A, Burtin C. Extra-pulmonary manifestations of COPD and the role of pulmonary rehabilitation: a symptom-centered approach. *Expert Rev Respir Med* 2021;15(1):131-42. doi: https://dx.doi.org/10.1080/17476348.2021.1854737
8. Chatila WM, Thomashow BM, Minai OA, et al. Comorbidities in chronic obstructive pulmonary disease. *Proc Am Thorac Soc* 2008;5(4):549-55. doi: 10.1513/pats.200709-148ET [published Online First: 2008/05/06]
9. Putcha N, Drummond MB, Wise RA, et al. Comorbidities and Chronic Obstructive Pulmonary Disease: Prevalence, Influence on Outcomes, and Management. *Semin Respir Crit Care Med* 2015;36(4):575-91. doi: 10.1055/s-0035-1556063 [published Online First: 2015/08/03]
10. Smith MC, Wrobel JP. Epidemiology and clinical impact of major comorbidities in patients with COPD. *Int J Chron Obstruct Pulmon Dis* 2014;9:871-88. doi: 10.2147/COPD.S49621
11. Maltais F, Decramer M, Casaburi R, et al. An official American Thoracic Society/European Respiratory Society statement: update on limb muscle dysfunction in chronic obstructive pulmonary disease. *Am J Respir Crit Care Med* 2014;189(9):e15-62. doi: 10.1164/rccm.201402-0373ST [published Online First: 2014/05/03]
12. Agusti AG. COPD, a multicomponent disease: implications for management. *Respir Med* 2005;99(6):670-82. doi: 10.1016/j.rmed.2004.11.006 [published Online First: 2005/05/10]
13. Vanfleteren LE, Spruit MA, Groenen M, et al. Clusters of comorbidities based on validated objective measurements and systemic inflammation in patients with chronic obstructive pulmonary disease. *Am J Respir Crit Care Med* 2013;187(7):728-35. doi: 10.1164/rccm.201209-1665OC
14. Agusti A, Calverley PM, Celli B, et al. Characterisation of COPD heterogeneity in the ECLIPSE cohort. *Respir Res* 2010;11(1):122. doi: 10.1186/1465-9921-11-122 [published Online First: 20100910]
15. Emtner M, Wadell K. Effects of exercise training in patients with chronic obstructive pulmonary disease--a narrative review for FYSS (Swedish Physical Activity Exercise Prescription Book). *Br J Sports Med* 2016;50(6):368-71. doi: 10.1136/bjsports-2015-095872 [published Online First: 2016/01/30]
16. Chang SS, Chen S, McAvay GJ, et al. Effect of coexisting chronic obstructive pulmonary disease and cognitive impairment on health outcomes in older adults. *J Am Geriatr Soc* 2012;60(10):1839-46. doi: 10.1111/j.1532-5415.2012.04171.x [published Online First: 2012/10/06]

For peer review only - http://bmjopen.bmj.com/site/about/guidelines.xhtml

17. Mostert R, Goris A, Weling-Scheepers C, et al. Tissue depletion and health related quality of life in patients with chronic obstructive pulmonary disease. *Respir Med* 2000;94(9):859-67.
18. Gosselink R, Troosters T, Decramer M. Peripheral muscle weakness contributes to exercise limitation in COPD. *Am J Respir Crit Care Med* 1996;153:976-80.
19. Hamilton AL, Killian KJ, Summers E, et al. Muscle strength, symptom intensity and exercise capacity in patients with cardiorespiratory disorders. *Am J Respir Crit Care Med* 1995;152:2021-31.
20. Coronell C, Orozco-Levi M, Mendez R, et al. Relevance of assessing quadriceps endurance in patients with COPD. *Eur Respir J* 2004;24(1):129-36. [published Online First: 2004/08/06]
21. Decramer M, Gosselink R, Troosters T, et al. Muscle weakness is related to utilization of health care resources in COPD patients. *Eur Respir J* 1997;10(2):417-23. [published Online First: 1997/02/01]
22. Gosselink R, Troosters T, Decramer M. Distribution of muscle weakness in patients with stable chronic obstructive pulmonary disease. *J Cardiopulm Rehabil* 2000;20(6):353-60. doi: 10.1097/00008483-200011000-00004 [published Online First: 2001/01/06]
23. Marquis K, Debigaré R, Lacasse Y, et al. Midthigh muscle cross- sectional area is a better predictor of mortality than body mass index in patients with chronic obstructive pulmonary disease. *Am J Respir Crit Care Med* 2002;166(6):809.
24. Swallow EB, Gosker HR, Ward KA, et al. A novel technique for nonvolitional assessment of quadriceps muscle endurance in humans. *J Appl Physiol (1985)* 2007;103(3):739-46. doi: 10.1152/japplphysiol.00025.2007 [published Online First: 2007/06/16]
25. Pedersen BK, Saltin B. Exercise as medicine - evidence for prescribing exercise as therapy in 26 different chronic diseases. *Scand J Med Sci Sports* 2015;25 Suppl 3:1-72. doi: 10.1111/sms.12581 [published Online First: 2015/11/26]
26. Spruit MA, Burtin C, De Boever P, et al. COPD and exercise: does it make a difference? *Breathe (Sheffield, England)* 2016;12(2):e38-e49. doi: 10.1183/20734735.003916
27. Wang T, Mao L, Wang J, et al. Influencing Factors and Exercise Intervention of Cognitive Impairment in Elderly Patients with Chronic Obstructive Pulmonary Disease. *Clin Interv Aging* 2020;15:557-66. doi: 10.2147/CIA.S245147
28. Maltais F, LeBlanc P, Jobin J, et al. Intensity of training and physiologic adaptation in patients with chronic obstructive pulmonary disease. *Am J Respir Crit Care Med* 1997;155(2):555-61. doi: 10.1164/ajrccm.155.2.9032194 [published Online First: 1997/02/01]
29. Fermont JM, Masconi KL, Jensen MT, et al. Biomarkers and clinical outcomes in COPD: a systematic review and meta-analysis. *Thorax* 2019;74(5):439-46. doi: 10.1136/thoraxjnl-2018-211855 [published Online First: 2019/01/09]
30. Gloeckl R, Marinov B, Pitta F. Practical recommendations for exercise training in patients with COPD. *European Respiratory Review* 2013;22(128):178. doi: 10.1183/09059180.00000513
31. Latimer LE, Constantin-Teodosiu D, Popat B, et al. Whole-body & muscle responses to aerobic exercise training and withdrawal in ageing & COPD. *Eur Respir J* 2021 doi: 10.1183/13993003.01507-2021 [published Online First: 20210929]
32. Rabinovich RA, Ardite E, Troosters T, et al. Reduced muscle redox capacity after endurance training in patients with chronic obstructive pulmonary disease. *Am J Respir Crit Care Med* 2001;164(7):1114-8. doi: 10.1164/ajrccm.164.7.2103065
33. Puente-Maestu L, Tena T, Trascasa C, et al. Training improves muscle oxidative capacity and oxygenation recovery kinetics in patients with chronic obstructive pulmonary disease. *Eur J Appl Physiol* 2003;88(6):580-7. doi: 10.1007/s00421-002-0743-9 [published Online First: 20021130]
34. Costes F, Gosker H, Feasson L, et al. Impaired exercise training-induced muscle fiber hypertrophy and Akt/mTOR pathway activation in hypoxemic patients with COPD. *J Appl Physiol (1985)* 2015;118(8):1040-9. doi: 10.1152/japplphysiol.00557.2014 [published Online First: 20150219]

For peer review only - http://bmjopen.bmj.com/site/about/guidelines.xhtml

35. Gouzi F, Préfaut C, Abdellaoui A, et al. Blunted muscle angiogenic training-response in COPD patients versus sedentary controls. *Eur Respir J* 2013;41(4):806. doi: 10.1183/09031936.00053512
36. Castagna O, Boussuges A, Vallier JM, et al. Is impairment similar between arm and leg cranking exercise in COPD patients? *Respir Med* 2007;101(3):547-53. doi: https://doi.org/10.1016/j.rmed.2006.06.019
37. Franssen FME, Wouters EFM, Baarends EM, et al. Arm mechanical efficiency and arm exercise capacity are relatively preserved in chronic obstructive pulmonary disease. *Med Sci Sports Exerc* 2002;34(10)
38. Shamseer L, Moher D, Clarke M, et al. Preferred reporting items for systematic review and meta-analysis protocols (PRISMA-P) 2015: elaboration and explanation. *BMJ* 2015;350:g7647. doi: 10.1136/bmj.g7647 [published Online First: 2015/01/04]
39. Page MJ, McKenzie JE, Bossuyt PM, et al. The PRISMA 2020 statement: an updated guideline for reporting systematic reviews. *BMJ* 2021;372:n71. doi: 10.1136/bmj.n71
40. Spruit MA, Singh SJ, Garvey C, et al. An official American Thoracic Society/European Respiratory Society statement: key concepts and advances in pulmonary rehabilitation. *Am J Respir Crit Care Med* 2013;188(8):e13-64. doi: 10.1164/rccm.201309-1634ST [published Online First: 2013/10/17]
41. Nyberg A, Probst V, Vaes AW. Assessments. In: Holland AE, Corso SD, Spruit MA, eds. Pulmonary Rehabilitation: European Respiratory Society 2021:360.
42. Higgins JPT TJ, Chandler J, Cumpston M, Li T, Page MJ, Welch VA (editors). Cochrane Handbook for Systematic Reviews of Interventions version 6.2 (updated February 2021): Cochrane, 2021, 2021.
43. Sterne JA, Savović J, Page MJ, et al. RoB 2: a revised tool for assessing risk of bias in randomised trials. *BMJ* 2019;366
44. Sterne JA, Hernán MA, Reeves BC, et al. ROBINS-I: a tool for assessing risk of bias in non-randomised studies of interventions. *BMJ* 2016;355
45. Downs SH, Black N. The feasibility of creating a checklist for the assessment of the methodological quality both of randomised and non-randomised studies of health care interventions. *J Epidemiol Community Health* 1998;52(6):377-84. doi: 10.1136/jech.52.6.377
46. Deeks JJ, Dinnes J, D'Amico R, et al. Evaluating non-randomised intervention studies. *Health Technol Assess* 2003;7(27):iii-x, 1-173. doi: 10.3310/hta7270
47. Frykholm E, Lima VP, Selander HV, et al. Physiological and Symptomatic Responses to Arm versus Leg Activities in People with Chronic Obstructive Pulmonary Disease: A Systematic Review and Meta-Analysis. *COPD* 2019;16(5-6):390-405. doi: 10.1080/15412555.2019.1674269 [published Online First: 20191021]
48. Hooper P, Jutai JW, Strong G, et al. Age-related macular degeneration and low-vision rehabilitation: a systematic review. *Can J Ophthalmol* 2008;43(2):180-87. doi: https://doi.org/10.3129/i08-001
49. Campbell M, McKenzie JE, Sowden A, et al. Synthesis without meta-analysis (SWiM) in systematic reviews: reporting guideline. *BMJ* 2020;368:l6890. doi: 10.1136/bmj.l6890
50. Maltais F, Decramer M, Casaburi R, et al. An official American Thoracic Society/European Respiratory Society statement: update on limb muscle dysfunction in chronic obstructive pulmonary disease. *Am J Respir Crit Care Med* 2014;189(9):e15-62. doi: https://dx.doi.org/10.1164/rccm.201402-0373ST

For peer review only - http://bmjopen.bmj.com/site/about/guidelines.xhtml

Reporting checklist for protocol of a systematic review and meta analysis.

Moher D, Shamseer L, Clarke M, Ghersi D, Liberati A, Petticrew M, Shekelle P, Stewart LA. Preferred Reporting Items for Systematic Review and Meta-Analysis Protocols (PRISMA-P) 2015 statement. Syst Rev. 2015;4(1):1.

		Reporting Item	Page
**Title**			
Identification	#1a	Identify the report as a protocol of a systematic review	1
Update	#1b	If the protocol is for an update of a previous systematic review, identify as such	-
**Registration**			
	#2	If registered, provide the name of the registry (such as PROSPERO) and registration number	2
**Authors**			
Contact	#3a	Provide name, institutional affiliation, e-mail address of all protocol authors; provide physical mailing address of corresponding author	1
Contribution	#3b	Describe contributions of protocol authors and identify the guarantor of the review	14
**Amendments**			
	#4	If the protocol represents an amendment of a previously completed or published protocol, identify as such and list changes; otherwise, state plan for documenting important protocol amendments	13
**-Support**			
Sources	#5a	Indicate sources of financial or other support for the review	13
Sponsor	#5b	Provide name for the review funder and / or sponsor	n/a
Role of sponsor or funder	#5c	Describe roles of funder(s), sponsor(s), and / or institution(s), if any, in developing the protocol	13
**Introduction**			
Rationale	#6	Describe the rationale for the review in the context of what is already known	5
Objectives	#7	Provide an explicit statement of the question(s) the review will address with reference to participants, interventions, comparators, and outcomes (PICO)	5 (6)
**Methods**			
Eligibility criteria	#8	Specify the study characteristics (such as PICO, study design, setting, time frame) and report characteristics (such as years considered, language,	5-7

For peer review only

		publication status) to be used as criteria for eligibility for the review	
Information sources	#9	Describe all intended information sources (such as electronic databases, contact with study authors, trial registers or other grey literature sources) with planned dates of coverage	7
Search strategy	#10	Present draft of search strategy to be used for at least one electronic database, including planned limits, such that it could be repeated	8 + appendix
Study records - data management	#11a	Describe the mechanism(s) that will be used to manage records and data throughout the review	8-9
Study records - selection process	#11b	State the process that will be used for selecting studies (such as two independent reviewers) through each phase of the review (that is, screening, eligibility and inclusion in meta-analysis)	8-9
Study records - data collection process	#11c	Describe planned method of extracting data from reports (such as piloting forms, done independently, in duplicate), any processes for obtaining and confirming data from investigators	8-9
Data items	#12	List and define all variables for which data will be sought (such as PICO items, funding sources), any pre-planned data assumptions and simplifications	9
Outcomes and prioritization	#13	List and define all outcomes for which data will be sought, including prioritization of main and additional outcomes, with rationale	9
Risk of bias in individual studies	#14	Describe anticipated methods for assessing risk of bias of individual studies, including whether this will be done at the outcome or study level, or both; state how this information will be used in data synthesis	9-11
Data synthesis	#15a	Describe criteria under which study data will be quantitatively synthesised	11-12
Data synthesis	#15b	If data are appropriate for quantitative synthesis, describe planned summary measures, methods of handling data and methods of combining data from studies, including any planned exploration of consistency (such as I2, Kendall's τ)	12
Data synthesis	#15c	Describe any proposed additional analyses (such as sensitivity or subgroup analyses, meta-regression)	12
Data synthesis	#15d	If quantitative synthesis is not appropriate, describe the type of summary planned	11
Meta-bias(es)	#16	Specify any planned assessment of meta-bias(es) (such as publication bias across studies, selective reporting within studies)	12
Confidence in cumulative evidence	#17	Describe how the strength of the body of evidence will be assessed (such as GRADE)	13

None The PRISMA-P elaboration and explanation paper is distributed under the terms of the Creative Commons Attribution License CC-BY. This checklist can be completed online using https://www.goodreports.org/, a tool made by the EQUATOR Network in collaboration with Penelope.ai

For peer review only

Database:
Ovid MEDLINE(R) and Epub Ahead of Print, In-Process, In-Data-Review & Other Non-Indexed Citations, Daily and Versions(R) <1946 to February 15, 2022>

Search as per 2022-02-15:

1. exp Pulmonary Disease, Chronic Obstructive/ (62214)
2. COPD.mp. (53231)
3. Chronic Obstructive Pulmonary Disease.mp. (55582)
4. Chronic Obstructive Airway Disease.mp. (309)
5. Chronic Obstructive Lung Disease.mp. (4419)
6. Airflow Obstruction$, Chronic.mp. (11)
7. Airflow limitation.mp. (3405)
8. exp Exercise/ (226020)
9. exp Exercise Therapy/ (58389)
10. Exercise Program.mp. (9485)
11. High-Intensity Interval.mp. (3443)
12. exp High-Intensity Interval Training/ (1691)
13. Interval Exercise.mp. (862)
14. Intermittent Exercise.mp. (1174)
15. Intermittent Training.mp. (191)
16. Continuous Exercise.mp. (788)
17. Continuous Training.mp. (1403)
18. Moderate-Intensity Continuous Exercise.mp. (173)
19. Sprint interval training.mp. (384)
20. Physical Exercise.mp. (18246)
21. Pulmonary Rehab*.mp. (4422)
22. Aerobic Exercise.mp. (11132)
23. Anaerobic exercise.mp. (622)
24. Exercise Training.mp. (19075)
25. exp Endurance Training/ (464)
26. Strength Training.mp. (5996)
27. exp Resistance Training/ (10819)
28. NMES.mp. (1389)
29. Neuromuscular Stimulation.mp. (525)
30. Inspiratory Muscle Training.mp. (831)
31. Cycling.mp. (70584)
32. Tai Chi.mp. (2083)
33. exp Tai Ji/ (1305)
34. exp Walking/ (62002)
35. 1 or 2 or 3 or 4 or 5 or 6 or 7 (97573)
36. 8 or 9 or 10 or 11 or 12 or 13 or 14 or 15 or 16 or 17 or 18 or 19 or 20 or 21 or 22 or 23 or 24 or 25 or 26 or 27 or 28 or 29 or 30 or 31 or 32 or 33 or 34 (374060)
37. 35 and 36 (6309)
38. exp animal/ not humans.sh. (4959724)
39. 37 not 38 (6264)

For peer review only

Modified 14-item Black and Downs checklist

1. Is the hypothesis/aim/objective of the study clearly described?
2. Are the main outcomes to be measured clearly described in the introduction or methods section?
3. Are the characteristics of the patients included in the study clearly described?
4. Are the distributions of principal confounders in each group of subjects to be compared clearly described?
5. Are the main findings of the study clearly described?
6. Does the study provide estimates of the random variability in the data for the main outcomes?
7. Have the characteristics of patients lost to follow-up been described?
8. Have actual probability values been reported for the main outcomes? (except where the probability value is <0.001)
9. Were the subjects asked to participate in the study representative of the entire population from which they were recruited?
10. Were those subjects who were prepared to participate representative of the entire population from which they were recruited?
11. Were the statistical tests used to assess the main outcomes appropriate?
12. Were the main outcome measures used accurate (valid and reliable)?
13. Were losses of patients to follow-up taken into account?
14. Did the study have sufficient power to detect a clinically important effect/difference where the probability value for a difference being due to chance is less than 5%?

For peer review only
